# Supplementary material for: Low-temperature strain-free encapsulation for perovskite solar cells and modules passing multifaceted accelerated ageing tests
Source: Nat Commun. 2024 May 29;15:4552. doi: 10.1038/s41467-024-48877-y (PMC11137052; doi:10.1038/s41467-024-48877-y)
Supplement: Supplementary file 1 — Supplementary Information [file 41467_2024_48877_MOESM1_ESM.docx]

**Supporting Information**

**Low-temperature strain-free semi-solid/liquid encapsulation for perovskite solar cells and modules passing multifaceted accelerated ageing tests**

*Paolo Mariani^ꝉ,1^, Miguel Molina García^ꝉ,2^, Jessica Barichello^1^, Marilena Isabella Zappia^2^, Erica Magliano^1^, Luigi Angelo Castriotta^1^, Luca Gabatel^2,3^, Sanjay Thorat^2^, Antonio Esaú Del Rio Castillo^2^, Filippo Drago^4^, Enrico Leonardi^5^, Sara Pescetelli^1^, Luigi Vesce^1^, Francesco Di Giacomo^1^, Fabio Matteocci^1^, Antonio Agresti^1^, Nicole De Giorgi,^2^ Sebastiano Bellani^ꝉ,2,*^ Aldo Di Carlo^1,6*^, and Francesco Bonaccorso^2,7*^*

*^ꝉ^ These authors contributed equally*

*^1^ CHOSE—Centre for Hybrid and Organic Solar Energy, University of Rome Tor Vergata, Via del Politecnico 1, 00133 Rome, Italy*

*^2^ BeDimensional S.p.A., Via Lungotorrente Secca 30R, 16163 Genova, Italy*

*^3^ Department of Mechanical, Energy, Management and Transport Engineering (DIME), Università di Genova, Genova, Italy*

*^4^ Nanochemistry Department, Istituto Italiano di Tecnologia, Via Morego 30, 16163 Genova, Italy*

*^5^ GreatCell Solar Italia SRL, Rome, Italy*

*^6^ ISM-CNR, Istitute of Structure of Matter, Consiglio Nazionale delle Ricerche, Rome, Italy*

*^7^ Graphene Labs, Istituto Italiano di Tecnologia, Via Morego 30, 16163 Genova, Italy*

Corresponding authors

Sebastiano Bellani: [s.bellani@bedimensional.it](mailto:s.bellani@bedimensional.it); Aldo Di Carlo: [aldo.dicarlo@uniroma2.it](mailto:aldo.dicarlo@uniroma2.it); Francesco Bonaccorso: [f.bonaccorso@bedimensional.it](mailto:f.bonaccorso@bedimensional.it)


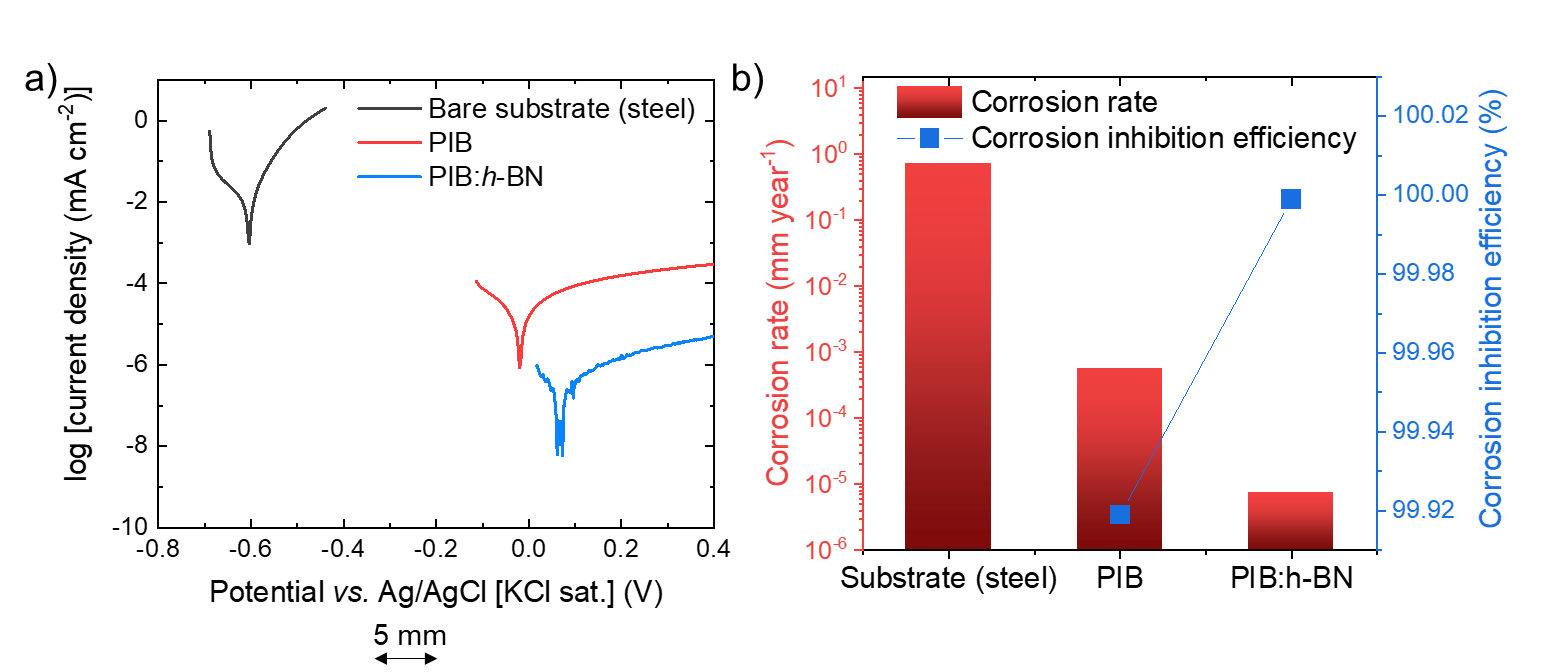


**Fig. S1.** a) Anodic polarisation curves (Tafel plots) of steel protected by solid (high-molecular weight) PIB and PIB:*h*-BN. The Tafel plot measured for bare steel is also shown for comparison. b) Corrosion rate of the investigated systems and the corrosion inhibition efficiency of the PIB and PIB:*h*-BN films. Data were reproduced from ref. 65 of the main text (Molina-Garcia, M. A. et al., *J. Phys. Mater*. **6**, 035006 (2023)). Solid PIB (Oppanol N80, average molecular weight 800,000) was purchased from BASF.


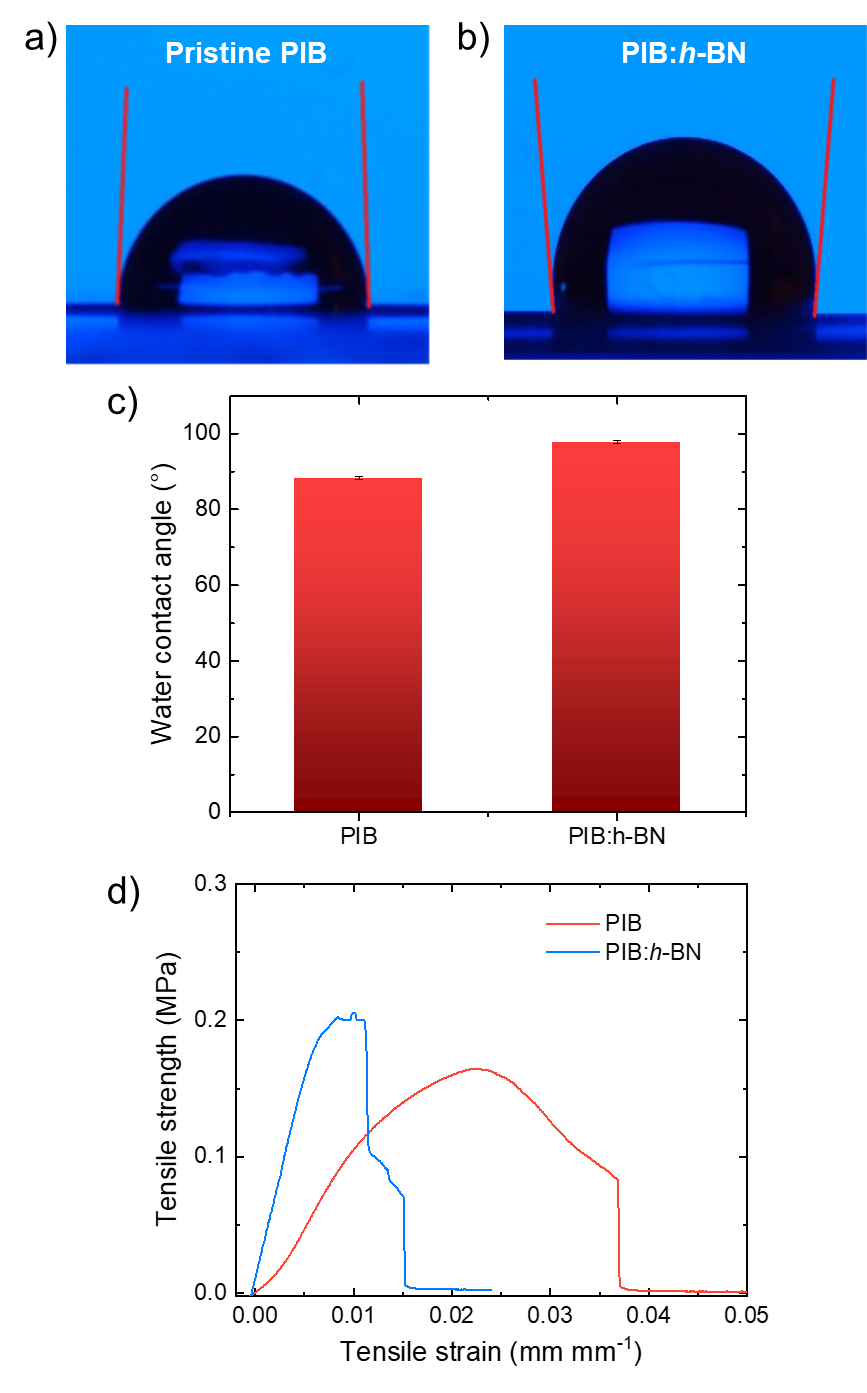


**Fig. S2**. Photographs of a water drop on the surface of the a) solid (high-molecular weight) PIB and b) PIB:*h*-BN films. c) Water contact angle data measured for the solid PIB and PIB:*h*-BN films. d) Tensile stress curves measured for solid PIB and PIB:*h*-BN films deposited on steel substrates. The films were produced with solid (high-molecular weight) PIB to avoid gravity-induced flatness alteration in viscoelastic films, which may lead to unreliable water contact angle results. Also, solid films for reliable pull-off measurements. Water contact angle data were reproduced from ref. 65 of the main text (Molina-Garcia, M. A. et al., *J. Phys. Mater*. **6**, 035006 (2023)). Solid PIB (Oppanol N80, average molecular weight 800,000) was purchased from BASF.


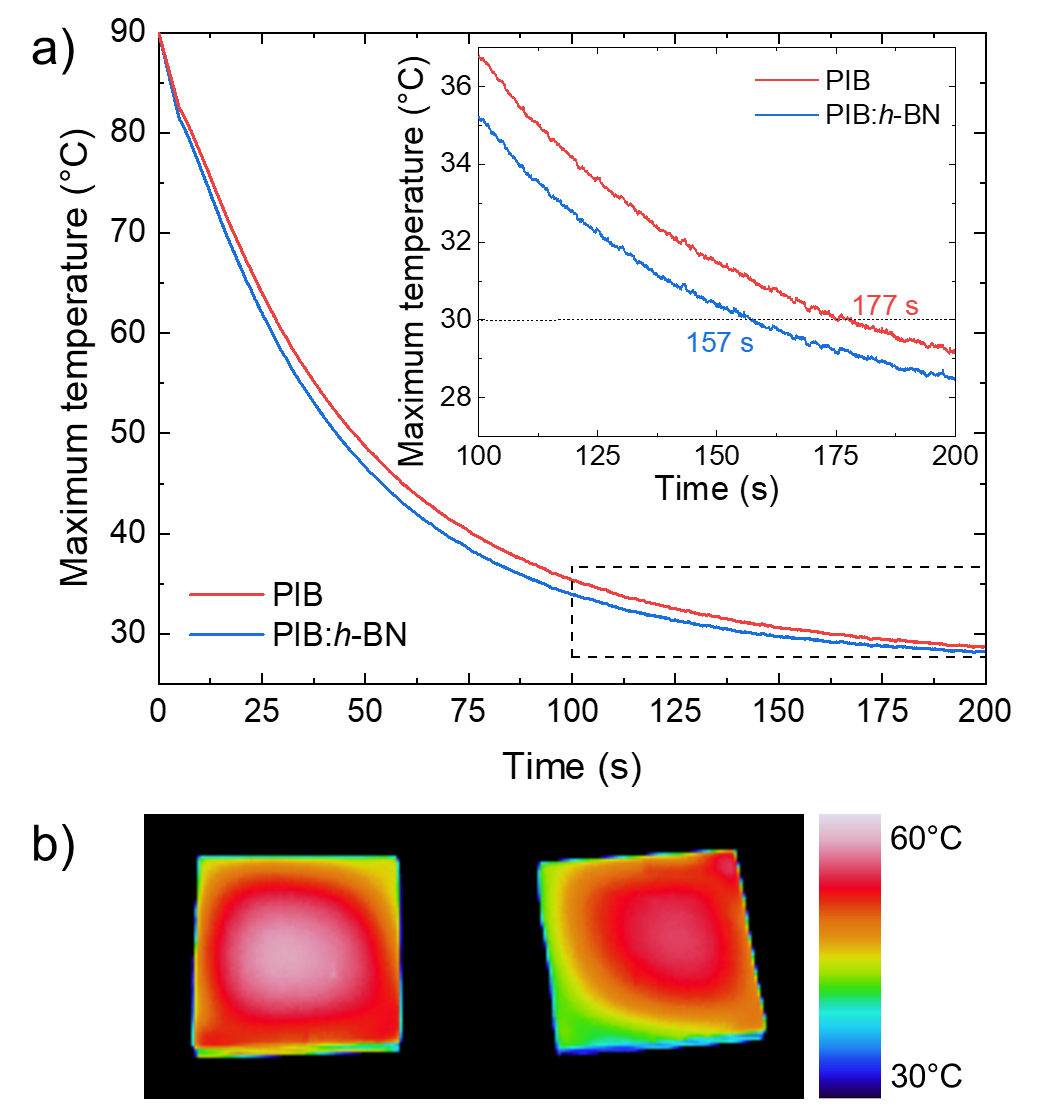


**Fig. S3**. a) Maximum temperature over time measured for the glass/PIB/glass and glass/PIB:*h*-BN/glass systems (area = 5.6 cm×5.6 cm) first heated at 90°C (t = 0 s) and then transferred to an Al platform at 25°C. The internal panel shows the magnification of the figure in the 100-200 s time interval. b) Infrared thermal images of glass/PIB/glass and glass/PIB:*h*-BN/glass systems acquired after 60 s of cooling.


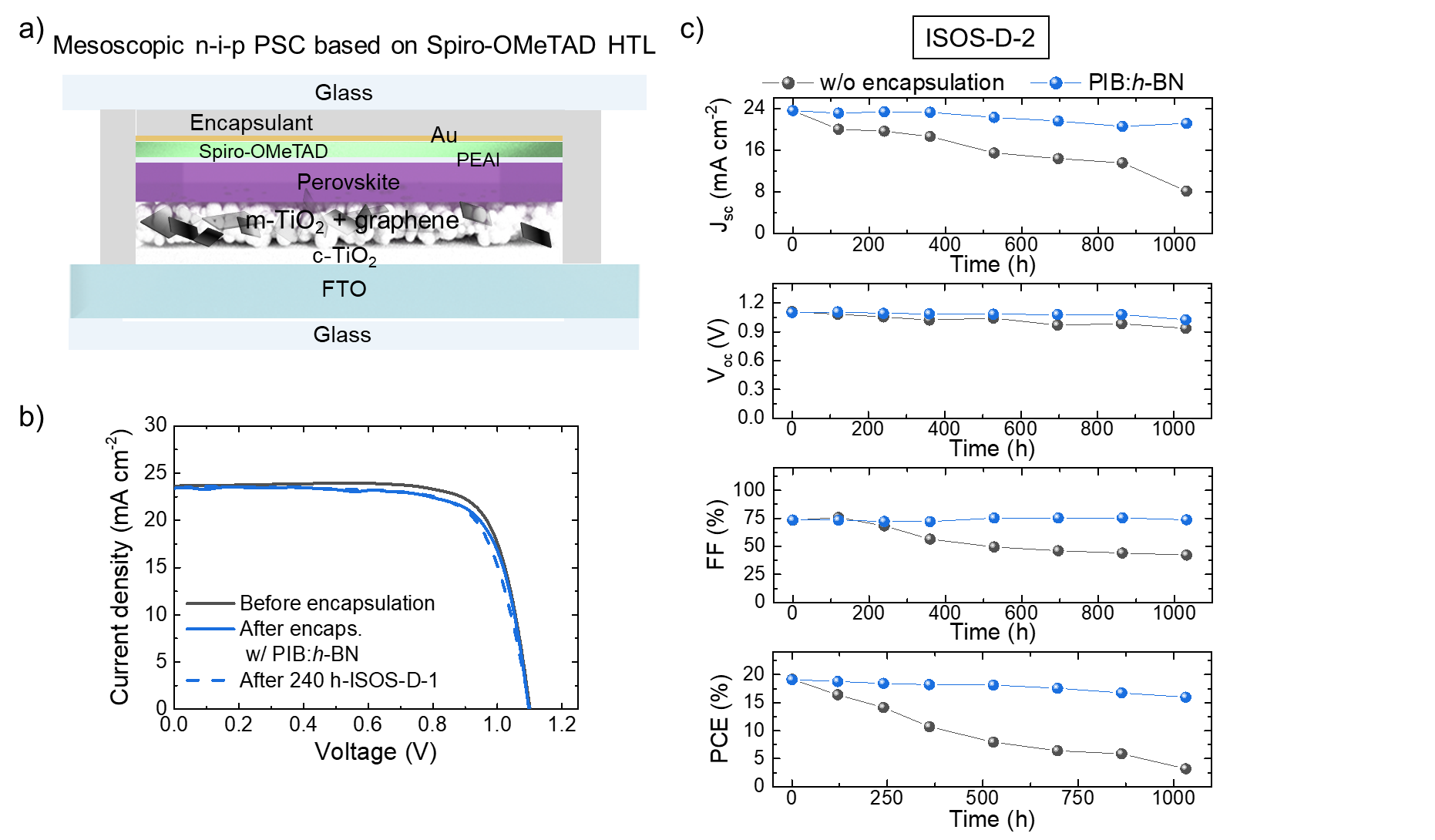


**Fig. S4.** a) Sketch of the structure of the large-area (1 cm^2^) mesoscopic n-i-p PSCs based on Cs_0.08_ FA_0.80_MA_0.12_Pb(I_0.88_ Br_0.12_)_3_ perovskites and spiro-OMeTAD HTLs. b) JV curves measured for the as-fabricated mesoscopic n-i-p PSCs based on spiro-OMeTAD HTLs before and after encapsulation with PIB:*h*-BN (before and after 240 h-ISOS-D-1). c) PV parameters of the investigated mesoscopic n-i-p PSCs based on spiro-OMeTAD HTLs acquired over >1000 h of ISOS-D-2 test.


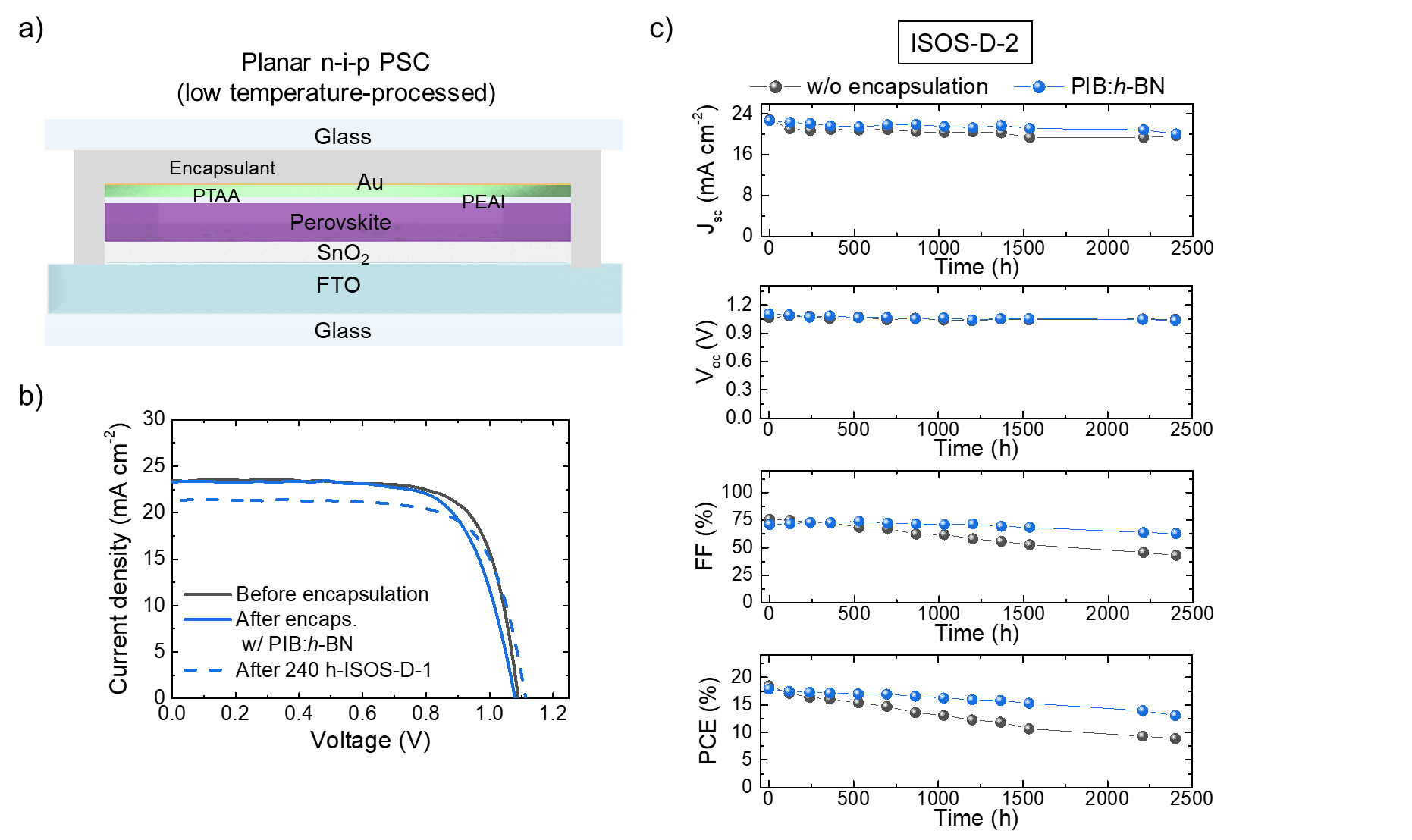


**Fig. S5.** a) Sketch of the structure of the low temperature-processed large-area (1 cm^2^) planar n-i-p PSCs based on Cs_0.08_ FA_0.80_MA_0.12_Pb(I_0.88_ Br_0.12_)_3_ perovskites. b) JV curves measured for the as-fabricated planar n-i-p PSCs before and after encapsulation with PIB:*h*-BN (before and after 240 h-ISOS-D-1). c) PV parameters of the investigated planar n-i-p PSCs acquired over >2000 h of ISOS-D-2 test.


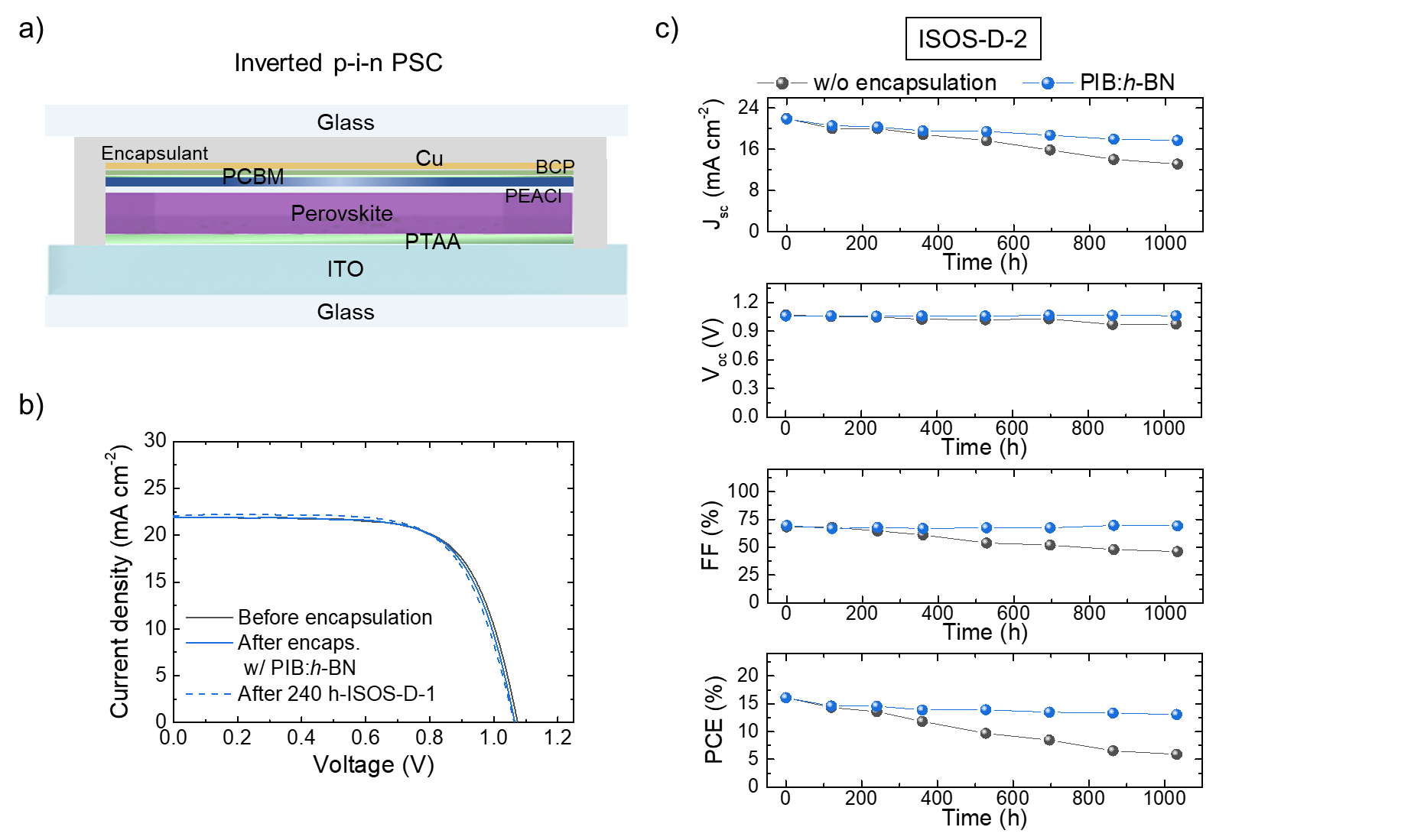


**Fig. S6.** a) Sketch of the structure of the large-area (1 cm^2^) inverted p-i-n PSCs based on PTAA HTLs and PCBM ETLs. b) JV curves measured for the as-fabricated inverted p-i-n PSCs before and after encapsulation with PIB:*h*-BN (before and after 240 h-ISOS-D-1). c) PV parameters of the investigated inverted p-i-n PSCs acquired over >1000 h of ISOS-D-2 test.


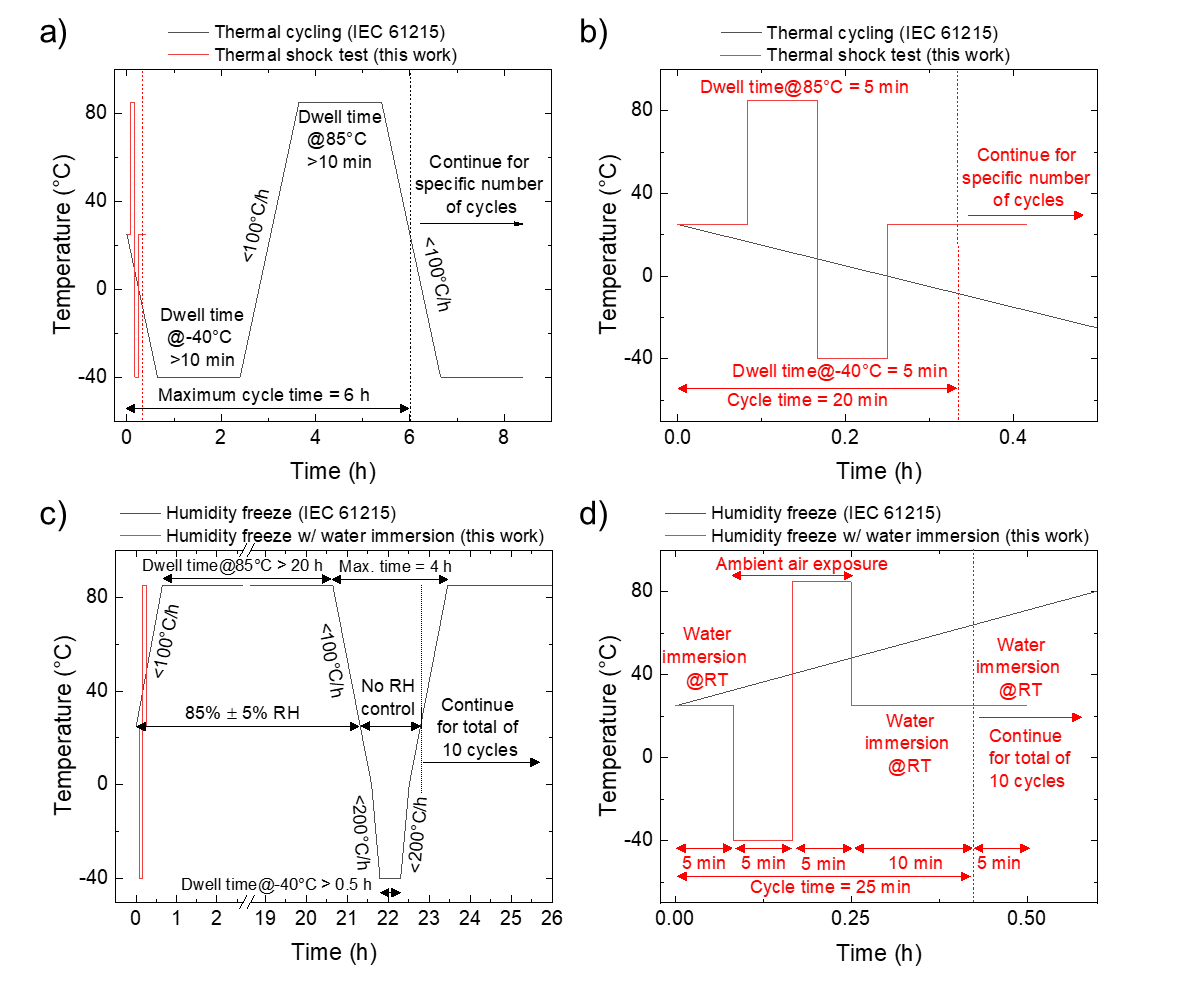


**Fig. S7.** a) Comparison between the temperature profiles of the IEC 61215 thermal cycling and our thermal shock test. b) Enlargement of the temperature profiles shown in panel a), evidencing the first cycle of our thermal shock test. c) Comparison between the temperature and RH profiles of the IEC 61215 and our humidity freeze tests. d) Enlargement of the temperature profiles and other environmental conditions (water immersion or ambient air exposure) shown in panel a), evidencing the first cycle of our humidity freeze test, including a water immersion step.


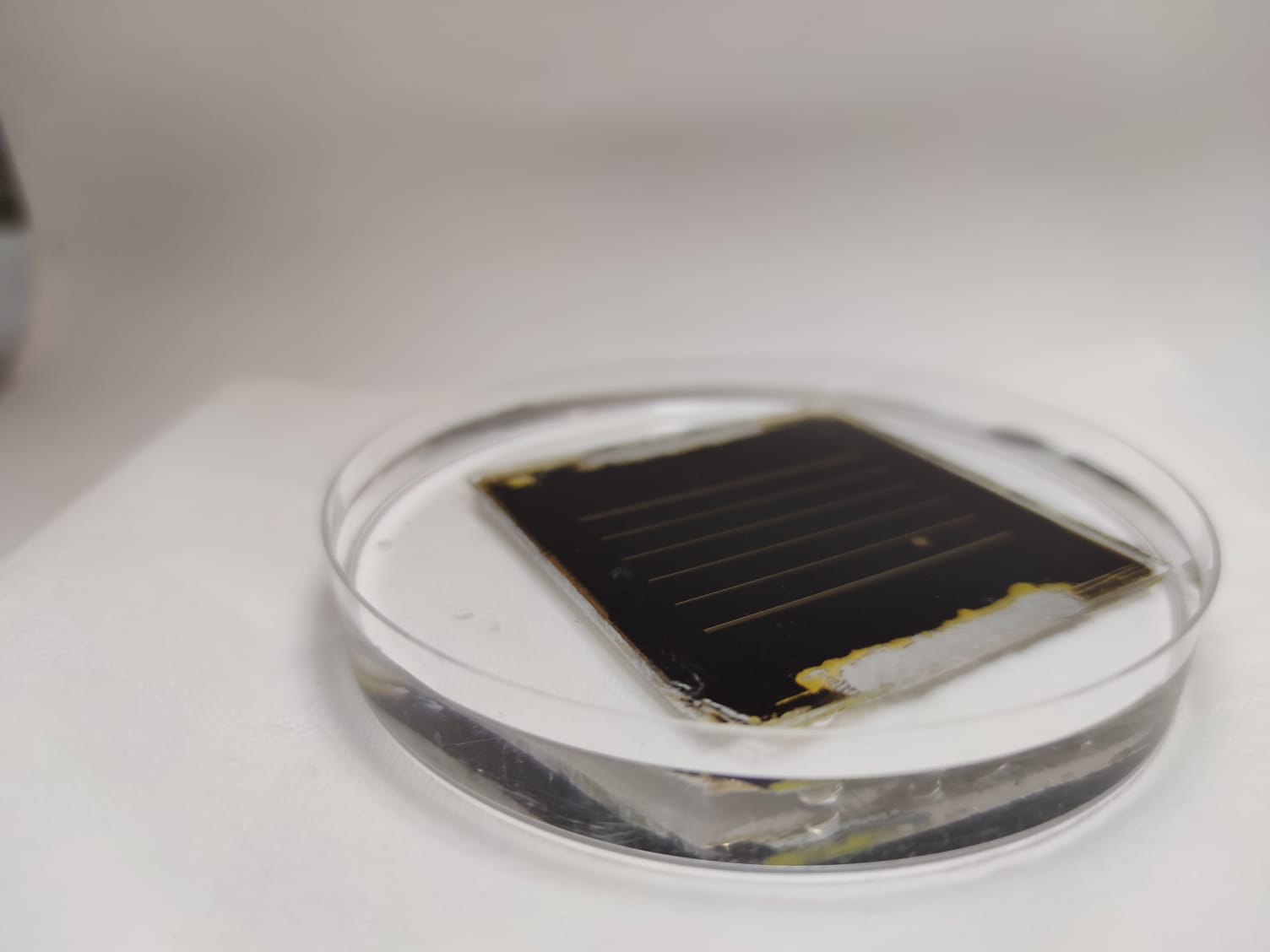


**Fig. S8.** Photograph of the PSM encapsulated with PIB:*h*-BN during the water immersion step of the modified humidity freeze test.

**
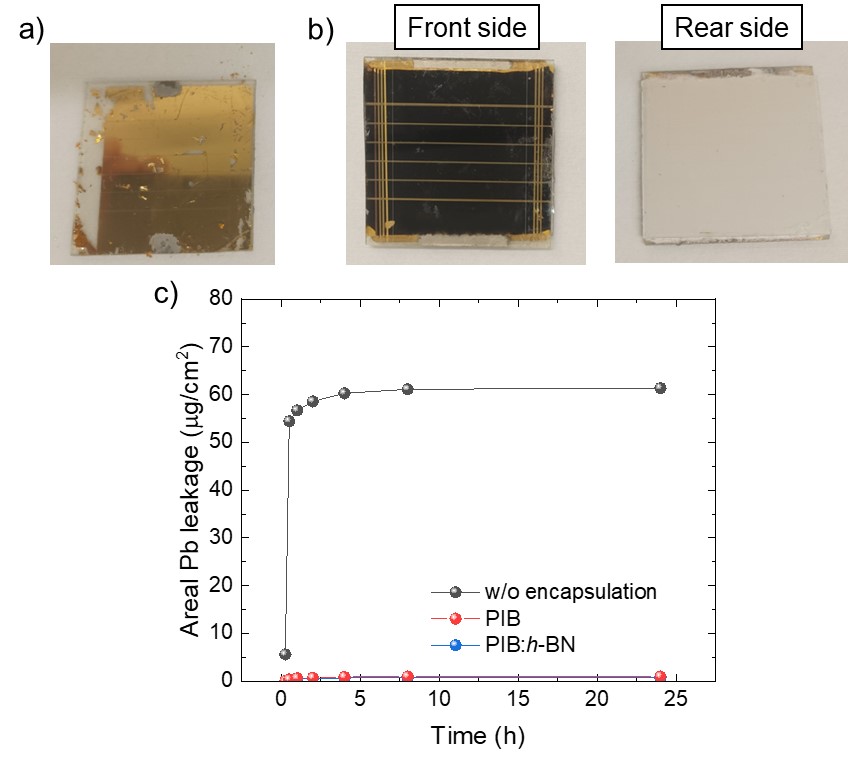
**

**Fig. S9.** a,b) Photograph of the unencapsulated PSM (rear side) and a PSM encapsulated with PIB:*h*-BN (rear and front side) after 24 h of immersion in water. c) Areal Pb leakage from the investigated PSMs over water immersion time. The Pb leakage from a perovskite film encapsulated with PIB was also measured (sample named PIB).


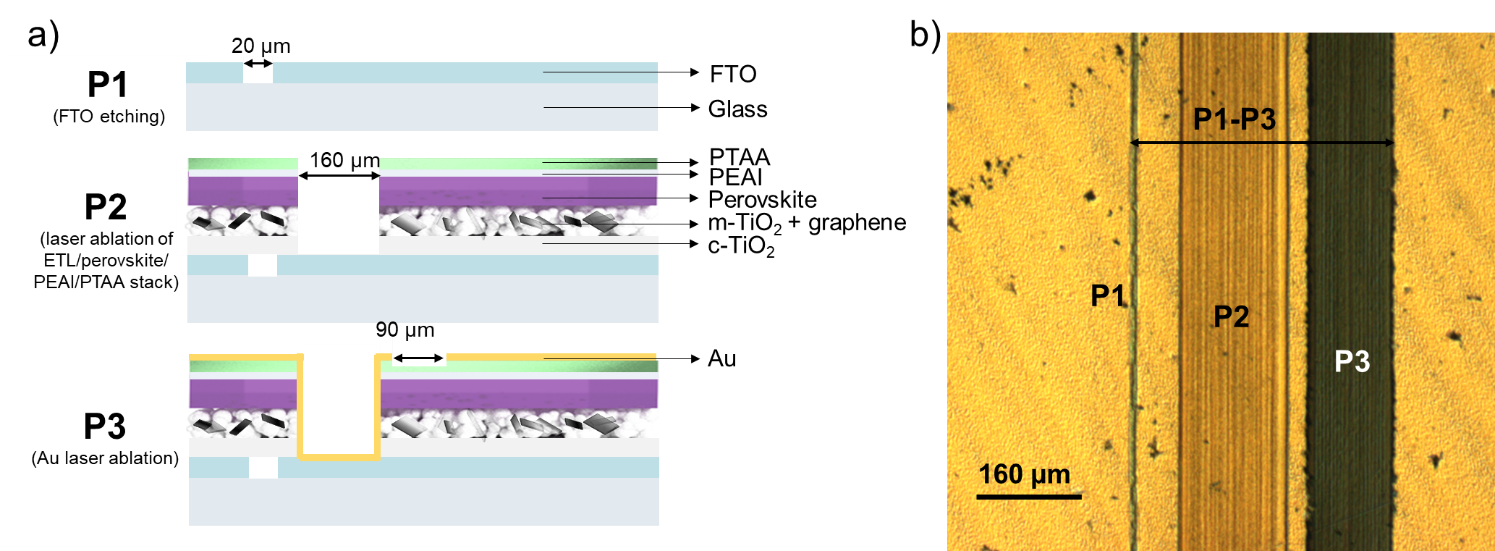


**Fig. S10.** a) Sketch of the laser ablation processes (P1-P2-P3) used to determine the PSM layout. b) Optical microscope image of the P1-, P2- and P3-ablated regions.

**Table S1**. PV parameters of the large-area (1 cm^2^) mesoscopic n-i-p PSCs based on PTAA HTLs, classified according to their encapsulants, tested through ISOS-D-2 protocols (after 240 h ISOS-D-1 test).

| **Encapsulant type** | **Status** | **Voltage scan mode** | **V_OC_**  **(V)** | **FF**  **(%)** | **J_SC_**  **(mA cm^-2^)** | **PCE**  **(%)** |
| --- | --- | --- | --- | --- | --- | --- |
| w/o encapsulation  (reference) | As-fabricated | Reverse | 1.08 | 74.6 | 22.85 | 18.37 |
|  |  | Forward | 1.05 | 67.7 | 22.60 | 16.05 |
|  | After 240 h ISOS-D-1 | Reverse | 1.08 | 75.2 | 22.03 | 18.15 |
|  |  | Forward | 1.09 | 68.3 | 21.69 | 16.09 |
| PIB | As fabricated and before encapsulation | Reverse | 1.08 | 75.4 | 23.00 | 18.71 |
|  |  | Forward | 1.11 | 71.1 | 22.73 | 17.88 |
|  | After encapsulation | Reverse | 1.07 | 74.3 | 22.64 | 18.06 |
|  |  | Forward | 1.07 | 70.2 | 22.99 | 17.23 |
|  | After 240 h ISOS-D-1 | Reverse | 1.07 | 72.9 | 22.72 | 17.33 |
|  |  | Forward | 1.07 | 74.2 | 21.48 | 16.96 |
| PIB:*h*-BN | As-fabricated and before encapsulation | Reverse | 1.11 | 72.7 | 22.99 | 18.61 |
|  |  | Forward | 1.11 | 70.9 | 22.60 | 17.86 |
|  | After encapsulation | Reverse | 1.10 | 70.8 | 23.36 | 18.11 |
|  |  | Forward | 1.09 | 72.8 | 21.68 | 17.13 |
|  | After 240 h ISOS-D-1 | Reverse | 1.10 | 71.4 | 22.72 | 17.98 |
|  |  | Forward | 1.08 | 71.1 | 21.97 | 17.06 |

**Table S2**. PV parameters of the large-area (1 cm^2^) mesoscopic n-i-p PSCs based on PTAA HTLs, classified according to their encapsulants, tested through ISOS-L-1 protocols (after 240 h ISOS-D-1 test).

| **Encapsulant type** | **Status** | **Voltage scan mode** | **V_OC_**  **(V)** | **FF**  **(%)** | **J_SC_**  **(mA cm^-2^)** | **PCE**  **(%)** |
| --- | --- | --- | --- | --- | --- | --- |
| **w/o encapsulation**  **(reference)** | As-fabricated | Reverse | 1.10 | 73.5 | 22.90 | 18.55 |
|  |  | Forward | 1.05 | 68.1 | 22.76 | 16.30 |
|  | After 240 h ISOS-D-1 | Reverse | 1.11 | 73.7 | 21.71 | 17.75 |
|  |  | Forward | 1.08 | 66.1 | 21.94 | 15.65 |
| **PIB** | As-fabricated and before encapsulation | Reverse | 1.11 | 73.4 | 23.13 | 18.78 |
|  |  | Forward | 1.05 | 68.2 | 23.15 | 16.64 |
|  | After encapsulation | Reverse | 1.10 | 72.8 | 22.52 | 18.01 |
|  |  | Forward | 1.06 | 65.6 | 22.92 | 16.01 |
|  | After 240 h ISOS-D-1 | Reverse | 1.10 | 73.8 | 21.87 | 17.72 |
|  |  | Forward | 1.04 | 62.9 | 22.14 | 14.45 |
| **PIB:*h*-BN** | As-fabricated and before encapsulation | Reverse | 1.10 | 73.6 | 22.79 | 18.50 |
|  |  | Forward | 1.05 | 68.9 | 22.81 | 16.56 |
|  | After encapsulation | Reverse | 1.09 | 74.4 | 22.26 | 18.11 |
|  |  | Forward | 1.04 | 66.5 | 22.26 | 15.41 |
|  | After 240 h ISOS-D-1 | Reverse | 1.10 | 73.2 | 22.24 | 17.95 |
|  |  | Forward | 1.05 | 63.2 | 21.84 | 14.46 |

**Table S3.** PV parameters of the large-area (1 cm^2^) mesoscopic n-i-p PSCs based on spiro-OMeTAD HTLs, classified according to their encapsulants, tested through ISOS-D-2 protocols (after 240 h ISOS-D-1 test).

| **Encapsulant type** | **Status** | **Voltage scan mode** | **V_OC_**  **(V)** | **FF**  **(%)** | **J_SC_**  **(mA cm^-2^)** | **PCE**  **(%)** |
| --- | --- | --- | --- | --- | --- | --- |
| w/o encapsulation  (reference) | As-fabricated | Reverse | 1.11 | 74.8 | 23.8 | 19.70 |
|  |  | Forward | 1.10 | 73.7 | 23.7 | 19.29 |
|  | After 240 h ISOS-D-1 | Reverse | 1.11 | 73.2 | 23.6 | 19..17 |
|  |  | Forward | 1.10 | 72.8 | 23.9 | 19.10 |
| PIB:*h*-BN | As-fabricated and before encapsulation | Reverse | 1.11 | 76.9 | 23.6 | 20.16 |
|  |  | Forward | 1.11 | 74.8 | 23.9 | 19.77 |
|  | After encapsulation | Reverse | 1.10 | 75.1 | 23.4 | 19.26 |
|  |  | Forward | 1.09 | 73.9 | 23.8 | 19.13 |
|  | After 240 h ISOS-D-1 | Reverse | 1.10 | 73.5 | 23.6 | 19.11 |
|  |  | Forward | 1.09 | 73.7 | 23.7 | 19.06 |

**Table S4.** PV parameters of the large-area (1 cm^2^) planar n-i-p PSCs based on low-temperature processed SnO_2_ ETLs, classified according to their encapsulants, tested through ISOS-D-2 protocols (after 240 h ISOS-D-1 test).

| **Encapsulant type** | **Status** | **Voltage scan mode** | **V_OC_**  **(V)** | **FF**  **(%)** | **J_SC_**  **(mA cm^-2^)** | **PCE**  **(%)** |
| --- | --- | --- | --- | --- | --- | --- |
| w/o encapsulation  (reference) | As-fabricated | Reverse | 1.08 | 75.8 | 23.12 | 18.95 |
|  |  | Forward | 1.00 | 74.8 | 22.83 | 18.86 |
|  | After 240 h ISOS-D-1 | Reverse | 1.07 | 75.7 | 22.86 | 18.43 |
|  |  | Forward | 1.07 | 72.4 | 23.59 | 18.27 |
| PIB:*h*-BN | As-fabricated and before encapsulation | Reverse | 1.09 | 73.6 | 23.46 | 18.87 |
|  |  | Forward | 1.10 | 75.1 | 22.27 | 18.35 |
|  | After encapsulation | Reverse | 1.09 | 71.2 | 23.28 | 18.00 |
|  |  | Forward | 1.09 | 72.2 | 22.80 | 18.00 |
|  | After 240 h ISOS-D-1 | Reverse | 1.11 | 72.7 | 21.44 | 17.35 |
|  |  | Forward | 1.09 | 69.7 | 22.76 | 17.05 |

**Table S5.** PV parameters of the large-area (1 cm^2^) inverted p-i-i PSCs based PTAA HTLs and PCBM ETLs, classified according to their encapsulants, tested through ISOS-D-2 protocols (after 240 h ISOS-D-1 test).

| **Encapsulant type** | **Status** | **Voltage scan mode** | **V_OC_**  **(V)** | **FF**  **(%)** | **J_SC_**  **(mA cm^-2^)** | **PCE**  **(%)** |
| --- | --- | --- | --- | --- | --- | --- |
| w/o encapsulation  (reference) | As-fabricated | Reverse | 1.08 | 68.7 | 22.0 | 16.3 |
|  |  | Forward | 1.06 | 69.3 | 22.1 | 16.3 |
|  | After 240 h ISOS-D-1 | Reverse | 1.07 | 68.3 | 22.0 | 16.1 |
|  |  | Forward | 1.07 | 68.1 | 22.0 | 16.0 |
| PIB:*h*-BN | As-fabricated and before encapsulation | Reverse | 1.08 | 69.2 | 22.1 | 16.6 |
|  |  | Forward | 1.07 | 69.9 | 21.9 | 16.4 |
|  | After encapsulation | Reverse | 1.07 | 69.6 | 21.9 | 16.3 |
|  |  | Forward | 1.06 | 69.6 | 21.9 | 16.3 |
|  | After 240 h ISOS-D-1 | Reverse | 1.06 | 69.6 | 21.9 | 16.1 |
|  |  | Forward | 1.06 | 69.1 | 22.0 | 16.0 |

**Table S6**. PV parameters of the mesoscopic PSMs, classified according to their encapsulants, tested through ISOS-D-2 protocols (after 240 h ISOS-D-1 test).

| **Encapsulant type** | **Status** | **Voltage scan mode** | **V_OC_**  **(V)** | **FF**  **(%)** | **J_SC_**  **(mA cm^-2^)** | **PCE**  **(%)** |
| --- | --- | --- | --- | --- | --- | --- |
| w/o encapsulation  (reference) | As-fabricated | Reverse | 5.25 | 74.5 | 19.34 | 15.13 |
|  |  | Forward | 5.03 | 67.2 | 22.19 | 15.00 |
|  | After 240 h ISOS-D-1 | Reverse | 5.05 | 74.2 | 20.09 | 15.06 |
|  |  | Forward | 5.05 | 63.9 | 21.94 | 14.16 |
| PIB | As-fabricated and before encapsulation | Reverse | 5.29 | 67.3 | 22.85 | 16.29 |
|  |  | Forward | 5.25 | 72.2 | 20.84 | 15.80 |
|  | After encapsulation | Reverse | 5.25 | 67.7 | 22.60 | 16.05 |
|  |  | Forward | 5.20 | 71.2 | 20.75 | 15.37 |
|  | After 240 h ISOS-D-1 | Reverse | 5.21 | 66.5 | 22.26 | 15.41 |
|  |  | Forward | 5.19 | 67.9 | 21.48 | 15.14 |
| PIB:*h*-BN | As-fabricated and before encapsulation | Reverse | 5.54 | 72.4 | 19.19 | 15.40 |
|  |  | Forward | 5.05 | 68.9 | 21.28 | 15.24 |
|  | After encapsulation | Reverse | 5.38 | 70.0 | 20.42 | 14.70 |
|  |  | Forward | 5.23 | 67.2 | 21.34 | 14.97 |
|  | After 240 h ISOS-D-1 | Reverse | 5.40 | 69.3 | 19.36 | 14.50 |
|  |  | Forward | 5.16 | 63.4 | 22.13 | 14.49 |

**Table S7**. PV parameters of the mesoscopic PSMs, classified according to their encapsulants, tested through ISOS-L-1 protocols.

| **Encapsulant type** | **Status** | **Voltage scan mode** | **V_OC_**  **(V)** | **FF**  **(%)** | **J_SC_**  **(mA cm^-2^)** | **PCE**  **(%)** |
| --- | --- | --- | --- | --- | --- | --- |
| **w/o encapsulation**  **(reference)** | As-fabricated | Reverse | 5.46 | 75.6 | 20.06 | 16.20 |
|  |  | Forward | 5.18 | 62.4 | 21.58 | 13.98 |
|  | After 240 h ISOS-D-1 | Reverse | 5.58 | 72.0 | 19.67 | 15.83 |
|  |  | Forward | 5.18 | 62.8 | 21.36 | 13.88 |
| **PIB** | As-fabricated and before encapsulation | Reverse | 5.40 | 75.2 | 20.99 | 17.03 |
|  |  | Forward | 5.36 | 63.3 | 22.68 | 15.41 |
|  | After encapsulation | Reverse | 5.39 | 72.7 | 20.83 | 16.31 |
|  |  | Forward | 5.20 | 61.0 | 22.73 | 14.44 |
|  | After 240 h ISOS-D-1 | Reverse | 5.22 | 75.1 | 20.66 | 16.18 |
|  |  | Forward | 5.22 | 57.5 | 21.80 | 13.07 |
| **PIB:*h*-BN** | As-fabricated and before encapsulation | Reverse | 5.20 | 76.9 | 20.52 | 16.86 |
|  |  | Forward | 5.33 | 63.7 | 20.59 | 13.97 |
|  | After encapsulation | Reverse | 5.10 | 73.6 | 21.39 | 16.01 |
|  |  | Forward | 5.36 | 60.5 | 22.10 | 13.85 |
|  | After 240 h ISOS-D-1 | Reverse | 5.21 | 75.4 | 20.27 | 15.93 |
|  |  | Forward | 5.19 | 62.4 | 21.12 | 13.68 |

**Table S8**. PV parameters of the mesoscopic PSMs, classified according to their encapsulants, tested through a customized thermal shock test (after 240 h ISOS-D-1 test). The cells were tested through a modified humidity freeze test after the thermal shock cycling.

| **Encapsulant type** | **Status** | **Voltage scan mode** | **V_OC_**  **(V)** | **FF**  **(%)** | **J_SC_**  **(mA cm^-2^)** | **PCE**  **(%)** |
| --- | --- | --- | --- | --- | --- | --- |
| PIB | As-fabricated and before encapsulation | Reverse | 5.29 | 67.4 | 22.85 | 16.28 |
|  |  | Forward | 5.24 | 68.1 | 22.20 | 15.84 |
|  | After encapsulation | Reverse | 5.26 | 66.7 | 22.50 | 15.77 |
|  |  | Forward | 5.27 | 68.9 | 21.45 | 15.57 |
|  | After 240 h ISOS-D-1 | Reverse | 5.30 | 67.2 | 21.90 | 15.62 |
|  |  | Forward | 5.18 | 68.6 | 21.27 | 15.12 |
| PIB:*h*-BN | As-fabricated and before encapsulation | Reverse | 5.32 | 69.5 | 21.95 | 16.23 |
|  |  | Forward | 5.29 | 68.9 | 21.24 | 15.51 |
|  | After encapsulation | Reverse | 5.21 | 71.6 | 21.33 | 15.94 |
|  |  | Forward | 5.19 | 67.6 | 22.06 | 15.49 |
|  | After 240 h ISOS-D-1 | Reverse | 5.29 | 69.0 | 21.24 | 15.50 |
|  |  | Forward | 5.25 | 66.2 | 21.69 | 15.07 |

**Table S9**. PV parameters of the large-area (planar) semi-transparent PSCs, before and after encapsulation with PIB encapsulants.

| **Cell type** | **Illumination side** | **Voltage scan mode** | **V_oc_**  **(V)** | **FF**  **(%)** | **J_sc_**  **(mA cm^-2^)** | **PCE**  **(%)** |
| --- | --- | --- | --- | --- | --- | --- |
| **w/o encapsulation** | Front | Reverse | 1.53 | 69.4 | 6.62 | 7.03 |
|  |  | Forward | 1.52 | 64.5 | 6.65 | 6.52 |
| **w/o encapsulation** | Rear | Reverse | 1.54 | 67.9 | 6.22 | 6.50 |
|  |  | Forward | 1.53 | 62.4 | 6.27 | 5.99 |
| **w/ encapsulation (PIB)** | Front | Reverse | 1.53 | 66.2 | 6.67 | 6.75 |
|  |  | Forward | 1.53 | 63.7 | 6.64 | 6.47 |
| **w/ encapsulation (PIB)** | Rear | Reverse | 1.54 | 63.6 | 6.14 | 6.01 |
|  |  | Forward | 1.55 | 63.6 | 6.18 | 6.09 |

**Table S10**. Parameters used for the laser ablation steps (P1, P2 and P3) during the fabrication of PSMs.

| **Step** | **Fluence per pulse**  **(mJ cm^-2^)** | **Plate speed (mm s^-1^)** | **Output power**  **(mW)** | **RSD** | **Width**  **(µm)** |
| --- | --- | --- | --- | --- | --- |
| **P1** | 648 | 195 | 595 | Single line | 20 |
| **P2** | 182 | 195 | 38 | 2x2 | 160 |
| **P3** | 206 | 195 | 43 | 3 | 90 |
