# Supplementary material for: Low-temperature strain-free encapsulation for perovskite solar cells and modules passing multifaceted accelerated ageing tests
Source: Nat Commun. 2024 May 29;15:4552. doi: 10.1038/s41467-024-48877-y (PMC11137052; doi:10.1038/s41467-024-48877-y)
Supplement: Supplementary file 3 — Reporting Summary [file 41467_2024_48877_MOESM3_ESM.pdf]

nature portfolio

Corresponding author(s): Francesco Bonaccorso

Solar Cells Reporting Summary

Nature Portfolio wishes to improve the reproducibility of the work that we publish. This form is intended for publication with all accepted papers reporting the characterization of photovoltaic devices and provides structure for consistency and transparency in reporting. Some list items might not apply to an individual manuscript, but all fields must be completed for clarity.

For further information on Nature Research policies, including our data availability policy, see Authors & Referees.

Experimental design

Please check the following details are reported in the manuscript, and provide a brief description or explanation where applicable.

1. Dimensions

Area of the tested solar cells

☒ Yes
☐ No

In the main text (both in the abstract and in the introduction), the active area of the devices is reported. Large area cells, active area: 1 cm2. The active area of a module is 10 cm2. The aperture area of the modules is 11 cm2 (i.e. geometrical fill factor = 91%) is reported in the main text ("Perovskite solar cell and module fabrication").

Explain why this information is not reported/not relevant.

Method used to determine the device area

☒ Yes
☐ No

The active area of a module is determined by laser patterning (Fig. 3a, Fig. S8, Table S8). The active area of large area cell was determined by a rectangular rigid plastic mask (1.6 cm x 0.63 cm).

Explain why this information is not reported/not relevant.

2. Current-voltage characterization

Current density-voltage (J-V) plots in both forward and backward direction

☐ Yes
☒ No

J-V plots are reported only in backward direction. For each device reported in the main text, photovoltaic parameters calculated from both forward and backward directions are reported in the Supporting Information.

Voltage scan conditions

☒ Yes
☐ No

For the large area cells, the voltage scan is 40 mV. This information is reported in the main text ("Device characterization"). Regarding the modules, the voltage scan is 400 mV.

Explain why this information is not reported/not relevant.

Test environment

☐ Yes
☒ No

Provide a description of the test conditions (e.g. characterization temperature, atmosphere, humidity).

The current-voltage characterization was perfomed in ambient condition. Consequently, no particular conditions were therefore specified in the main text.

Protocol for preconditioning of the device before its characterization

☐ Yes
☒ No

Provide a description of the protocol.

We stabilized the Voc of each measured device for 30 s before the measurements. This information is not relevant since it is a protocol commonly established in previous publications.

Stability of the J-V characteristic

☒ Yes
☐ No

Maximum power point tracking. This information is reported in the main text ("Device characterization")

Explain why this information is not reported/not relevant.

3. Hysteresis or any other unusual behaviour

Description of the unusual behaviour observed during the characterization

☒ Yes
☐ No

In the Supporting Information, we reported PCE and other photovoltaic metric data, showing a variable hysteresis. This behaviour is often observed for perovskite solar cells.

Explain why this information is not reported/not relevant.

Related experimental data

☒ Yes
☐ No

Tables S1, S2, S3, S4 and S5 in the Supporting Information

Explain why this information is not reported/not relevant.

4. Efficiency

External quantum efficiency (EQE) or incident photons to current efficiency (IPCE)

☐ Yes
☒ No

Provide a description of the technique used.

These data were not relevant for the message of this work, focusing on stabilizing the performances of large-area cells and modules without state-of-the-art performances. Not supported for large area devices.

A comparison between the integrated response under the standard reference spectrum and the response measure under the simulator

☒ Yes
☐ No

All the devices are measured under calibrated Class-A Sun Simulator equipped with an AM1.5G filter. This information is reported in the main text ("Device characterization")

Explain why this information is not reported/not relevant.

For tandem solar cells, the bias illumination and bias voltage used for each subcell

☐ Yes
☒ No

Provide a description of the measurement conditions.

This work does not report any tandem solar cell.

5. Calibration

Light source and reference cell or sensor used for the characterization

☒ Yes
☐ No

J-V measurements of the devices were performed with a Class-A Sun Simulator equipped with an AM1.5G filter. The sun simulator was calibrated to 1 Sun illumination condition with a Si-based reference cell (RR-226-O, RERA Solutions). This information is reported in the main text ("Device characterization")

Explain why this information is not reported/not relevant.

Confirmation that the reference cell was calibrated and certified

☐ Yes
☒ No

Identify the independent certification laboratory.

We have taken this information for granted as the reference cell is regularly calibrated in our laboratories where solar cells and modules are characterized.

Calculation of spectral mismatch between the reference cell and the devices under test

☐ Yes
☒ No

Provide a value of the spectral mismatch and/or a description of how it has been taken into account in the measurements.

We have taken this information for granted as this calculation is automatically done by our equipment software to accurately report characterization data of solar cells and modules.

6. Mask/aperture

Size of the mask/aperture used during testing

☐ Yes
☒ No

Report the size of the mask/aperture.

During the stabilty tests, the mask of the large area cells is the one adopted for the JV characterization (a rectangular rigid plastic mask (1.6 cm x 0.63 cm)). During the stabilty tests, the modules are not masked. Since no particular practices were used, this information was not reported in the manuscript.

Variation of the measured short-circuit current density with the mask/aperture area

☐ Yes
☒ No

Report the difference in the short-circuit current density values measured with the mask and aperture area.

As can be understood from the information reported in the text, the active area dimension for the moduels is fixed by the laser scribing process. For the large-area cells, the active area dimension was fixed by the rectangular rigid plastic mask. It was not our purpose to check data on active area smaller than those resembling the entire active area of the device.

7. Performance certification

Identity of the independent certification laboratory that confirmed the photovoltaic performance

☐ Yes
☒ No

Identify the independent certification laboratory.

We did not certify the photovoltaic performance because none record was reported (e.g., in terms of PCE).

A copy of any certificate(s)

☐ Yes
☒ No

Certificate copies should be provided in the Supplementary information. Please state the supplementary item number.

We did not certify the photovoltaic performance because none record was reported (e.g., in terms of PCE).

8. Statistics

Number of solar cells tested

☒ Yes
☐ No

For the most representative cells and modules, at least three devices have been tested, in some cases both before and after encapsulation.

Explain why this information is not reported/not relevant.

Statistical analysis of the device performance

☐ Yes
☒ No

State where this information can be found in the text.

This information was not relevant for the purpose of this work, which instead focused on the performance stability of devices under multifaceted accelerated ageing tests that ensured data reliability.

9. Long-term stability analysis

Type of analysis, bias conditions and environmental conditions

☒ Yes
☐ No

We performed different accelerated ageing tests, including ISOS-D1 preconditioning (240 h), ISOS-D2 (85°C, >1000 h), ISOS-L1 (light soaking, >1000 h), as well as a customized thermal shock test (200 cycles) and modified humidity freeze test (10 cycles). These informations are reported in several parts of the main text

Explain why this information is not reported/not relevant.
